# Effect of the Addition of Donkey Milk on the Acceptability of Caciotta Cow Cheese

**DOI:** 10.3390/ani12111444

**Published:** 2022-06-03

**Authors:** Carlo Cosentino, Rosanna Paolino, Mariarita Rubino, Pierangelo Freschi

**Affiliations:** School of Agricultural, Forestry, Food and Environmental Sciences (SAFE), University of Basilicata, 85100 Potenza, Italy; carlo.cosentino@unibas.it (C.C.); rubino.mariarita96@gmail.com (M.R.); pierangelo.freschi@unibas.it (P.F.)

**Keywords:** short-ripened cheese, innovative product, high value markets

## Abstract

**Simple Summary:**

Caciotta is a very popular cheese in Italy that can be made with cow, sheep, goat or buffalo milk. In this study, we investigated the effects of donkeys’ milk addition in the production of this cheese, considering a short- and a medium-ripening period. Two batches were considered for cheesemaking: cows’ milk only and cows’ milk with the addition of 5% donkey’s milk. With the longest ripening period, the cheese with donkey milk received the highest ratings for color, aroma, flavor, and overall liking.

**Abstract:**

This study investigated the effects of adding donkey milk in cheesemaking on the acceptability of a Caciotta cow cheese after 10 and 45 days of ripening. The cheeses produced were: a control cheese with cow’s milk only and experimental cheese with the addition of 5% donkey’s milk. The acceptability of Caciotta was determined by the judgement of 80 habitual cheese consumers. The acceptability of the Caciotta cheese was significantly influenced by the addition of donkey milk, with the exception of the texture parameter. At a ripening time of 10 days, the control cheese scored significantly higher than the experimental cheese for aroma, flavor and overall liking; conversely, at the longest time, the experimental cheese had significantly higher scores for color, aroma, flavor, and overall liking. Our results confirm that the use of donkey milk in cheesemaking can improve cheese acceptability. In addition, the known benefits of using donkey milk in cheesemaking, such as the reduction of blowing defects and the probiotic properties, could increase interest in innovative products among both processors and consumers. Processors could reduce, if not eliminate, the use of additives in cheesemaking, while cheese could also appeal to consumers of probiotic and fermented products.

## 1. Introduction

Caciotta is a fresh or semi-hard cheese with a short (15–20 days) or long (2–6 months) maturation [1], whose production in Italy is about 23,000 tons per year [2]. This type of cheese is found in many geographical areas of Italy but is similar to other local cheeses [3] with different sensory characteristics, which also depend on the milk used: cow, sheep, goat, and buffalo milk. It is often made from a mixture of different types of milk, as they have different flavor profiles: cow’s milk is known to contain the least flavoring. In Tuscany, Caciotta is made mainly from cow’s milk, and a small amount is made from sheep’s milk, and it is probably the best-known variety in Italy. It has a sweet taste and a milky white paste, while the rind is usually straw-colored. In the Siena area, it is traditional to color the rind with tomatoes, while in the past even lamb’s blood was used. In Latium, Caciotta romana is made from sheep’s milk and has a soft, straw-white color and delicate taste. It can be eaten fresh, in which case it is called “primosale”, or it is left to mature for about fifteen days. In the Marche region, on the other hand, the Protected Designation of Origin “Casciotta d’Urbino” is made from 70% sheep’s milk and 30% cow’s milk; it has a sweet, slightly acidic milk taste, and its texture is soft and crumbly [4]. Caciotta made from goat’s milk is produced in both Campania and Sicily and has a very strong flavor due to the goat’s milk, giving it notes of wild plants. Caciotta made from buffalo milk is produced in the regions of Latium and Campania and has the typical aftertaste of buffalo milk, slightly acidic but sweet; its paste is white, and the texture is soft and compact. Caciotta cheese is made from pasteurized or raw whole milk and is usually inoculated with thermophilic and/or mesophilic lactic acid bacteria. It has a cylindrical shape (4 to 8 cm high and 8 to 16 cm in diameter), and weighs 0.8 to 2.0 kg, usually 1 kg, with a dark ivory crust and a lighter compact interior [5]. The sensory characteristics of Caciotta depend on the biochemical and microbiological activities that follow each other during ripening. At this stage, the sensory quality of Caciotta can be altered by aromas resulting from undesirable fermentation and by excessive concentrations of bitter oligopeptides [6]. The microbiota of cheese come from three sources: the native milk microbiota, the inoculated enzymes, and the cheesemaking environment [7]. A fundamental aspect affecting the microbiota is hygiene throughout the supply chain. Nowadays, different approaches are used to reduce the microbial load in order to ensure the safety of milk and cheese. Calasso et al. [6] demonstrated that the use of attenuated starters has a strong impact on the microbiota of Caciotta cheese. Bancalari et al. [1] found that the addition of an adjuvant aromatic culture, *Lactobacillus paracasei* 4341, during cheesemaking improved the aroma profile and the physicochemical and rheological properties of a medium-aged Caciotta cheese. Previous studies have tested donkey milk, used in percentages between 1 and 10%, as an inhibitor of late swelling by clostridia and coliforms in semi-hard ewes’ and goats’ milk cheeses [8,9,10], as well as in cheesemaking for innovative products [1,11,12] and in the production of fermented milk and innovative beverages [13]. Donkey milk inhibits the effect on microorganisms in a semi-hard cow milk cheese [10] such as in Grana [14] and Turkish Kashar cheese [15]. To date, no study has described the consumer response to a mixed Caciotta cheese made from cows’ and donkeys’ milk. In a similar semi-hard cow’s milk cheese with the addition of 7.5% donkey milk, Cosentino et al. [9] observed a significant reduction in swelling but no effect on acceptability. Paolino et al. [8] used two additions of donkey milk, 5 and 10%, in the production of a goat cheese and showed that in 45 days ripened cheese, the lower addition significantly improved some acceptance parameters.

Based on the above results, in this preliminary study we evaluated some acceptability parameters of Caciotta cheese considering the addition of 5% donkey milk and a ripening period of 10 and 45 days. The aim of this work was to make a preliminary evaluation of the possibility of combining the traditional production of Caciotta, which is highly appreciated on the national market in all its regional specificities, with an innovative product without additives such as egg lysozyme, which can cause severe reactions in consumers who are allergic to eggs.

## 2. Material and Methods

### 2.1. Milk Collection and Proximate Analysis

Milk was collected on the same day from pluriparous Fresian cows and Martinafranca donkeys in two farms with mechanical milking machines and then refrigerated at 4 °C. The milk was taken to the laboratory to determine the protein, fat, and lactose content by Milkoscan (Milkoscan 133-B, Foss Electric, Hillerod, Denmark) according to the International Organization for Standardization (ISO) procedures [16], using the corresponding primary reference samples from the standard milk laboratory of the Italian Breeders’ Association. The dry matter (DM) and ash analysis [17] and pH (ph meter HI931410; Hanna Instruments, Italy) were determined. The analyses were performed in duplicate.

### 2.2. Cheesemaking

The Caciotta cheese made from cow and donkey milk was produced at a dairy farm in the Basilicata region, using a slight variation of the cheesemaking process described by Cosentino et al. [10]. The cow’s milk was first pasteurized at 65 °C for 30 min and then cooled to 42 °C. Donkey milk was not thermized because heat treatment alters the conformation of the protein, leading to negative results in the enzymatic reaction [18]. Cow milk was inoculated at 42 °C with lyophilized lactic ferments of *Streptococcus thermophilus* (6 g/hL; FL 058, Prodor, Piacenza, Italy). After 15 min at 39 °C, a fixed volume of pasteurized cow’s milk (100 L for each container) was poured into 2 different containers to account for two different theses: control cheese (cow’s milk only), and experimental cheese (5% donkey’s milk). At 37 °C, liquid calf rennet (30 mL/hL; strength units 1:10,000; ratio of chymosin to pepsin 80:20, Santamaria Srl, Burago di Molgora, Italy) was added to the milk, and curds were obtained in each group within 30 min. After 45 min at 36 °C, the curd was cut from each vat (particle diameter 2 cm) and then broken into the size of rice grains (0.5 cm). The serum was removed, and the curd was pressed into cylindrical moulds (diameter = 14 cm, height = 8 cm, weight = 1 kg) and then stewed at 36 °C to a pH of 5.4. After draining the whey (2 h), the Caciotta cheeses were dry-salted (NaCl, pH 5.20) and then stored at 8–10 °C for 10 and 45 days. The whole experiment was repeated twice.

The Caciotta cheeses were sampled according to International Dairy Federation (IDF) standards [19] to determine the following parameters: pH (pHmeter HI98161, Hanna Instruments, Padova, Italy), protein, fat (Milkoscan 133-B, Foss Electric, Hillerod, Denmark), DM and ash content [17]. The analyses were performed in duplicate.

### 2.3. Consumer Acceptability

The acceptability of control and experimental cheese after 10 and 45 days of ripening was determined by the judgement of habitual cheese consumers (41 men and 39 women, aged 21–58 years) recruited through advertizing on the campus of the University of Basilicata. Before the test, a short training session was conducted to familiarize consumers with the descriptors, and they had no information about the experimental design. They were asked to evaluate the sensory parameters described and to use the water and unsalted crackers available to them. A small amount (20 g) of each Caciotta was labeled with a random 3-digit code, left at room temperature for 1 h, and then served to consumers in a random order under white fluorescent light together with a sensory evaluation questionnaire. They were asked to rate the color, aroma, flavor, texture and overall liking using a 9-point hedonic scale [3,10]. The consumer test was conducted in individual booths.

### 2.4. Statistical Analysis

The homogeneity of variances was tested using Levene’s test. The data were subjected to ANOVA using a bifactorial model that included the type of cheese (control cheese and experimental cheese) and the ripening time (10 and 45 days). The interaction between the factors was not significant. The mean values were compared with Tukey’s HSD, and *p* < 0.05 was accepted as the significance level.

## 3. Results

Cow milk contained on average of 3.31% protein, 4.48% fat, 4.53% lactose, 12.77% DM, and 0.88% ash, pH 6.77 at 25 °C. Donkey milk had the following composition: 1.51% protein, 0.32% fat, 6.37% lactose, 8.80% DM, and 0.40% ash content, pH 7.15 at 25 °C.

The comparison of the proximate composition between control and experimental cheeses showed no significant differences in dry matter, protein, fat, and ash content in both ripening periods, while pH values decreased significantly, *p* < 0.05 and *p* < 0.001, from 10 to 45 days of ripening for control cheese and experimental cheese (Table 1).

The acceptability of the Caciotta cheese was significantly influenced by the addition of donkey milk at 10 days (*p* < 0.001) and at 45 days (*p* < 0.05), except for the texture parameter (Table 2). The overall liking of the four kinds of cheeses was above six points (6 = slightly pleasant) on the 9-point hedonic scale. 

In particular, at 10 days of ripening, control cheese scored significantly higher than experimental cheese (*p* < 0.05) for aroma (7.13 vs. 6.64), flavor (7.27 vs. 6.62), and overall liking (7.29 vs. 6.71), while at the longest ripening, experimental cheese was preferred for aroma (7.11 vs. 6.64) (*p* < 0.05), and (P < 0.01) for color (7.38 vs. 6.76), flavor (7.51 vs. 6.87), and overall liking (7.56 vs. 6.87).

The cheese made with cow’s milk only was preferred at the lower ripening time, except for the texture parameter. In particular, significant (*p* < 0.05) differences were found for color (+0.48) and aroma (+0.49) compared to the ripening time of 45 d. Conversely, for the experimental cheese, all acceptance parameters improved with the longest ripening time, with significantly (*p* < 0.001) higher values for flavor (+0.89) and overall liking (+0.85) compared to the shortest ripening time (Table 2).

## 4. Discussion

The study showed that the addition of a small amount in the production of Caciotta with a mead-ripening period would allow for an even more pleasant product, which would probably also have a longer shelf life due to the bacteriostatic effect of donkey milk [10]. Conversely, the lowest rating for flavor and overall liking in the medium-ripened control cheese could be explained by a lower bacterial inhibition due to the absence of donkey milk. Other studies on mixed goat- [8,20] and cow-donkey cheeses [9,14] have shown that mixed cheeses are better accepted, at certain ripening times, than cheeses made only from goat or cow milk. This was observed with Grana Padano, the famous aged hard cheese [14], and with a semi-hard cheese similar to Caciotta but made with kid rennet [9], where the different temperatures and ripening times had no effect on acceptability. Considering more closely the acceptance parameters, the color parameter for the mixed milk cheese improved significantly after 45 days of ripening, similar to a recent study [8] where consumers preferred the color of the mixed goat/donkey cheese to that of the goat-only control cheese. In a cheese made exclusively from donkeys’ milk, the brightness affecting the color attribute was found to be related to the moisture content and a lower number of free drops, resulting in less light dispersion [21]. The acceptability of the aroma and flavor parameters was significantly improved at the end of ripening, similar to mixed cow/donkey milk cheeses from Italy [10,20] and Turkey [15]. In the ripening process, a general increase in flavor is associated with the production of a wide range of volatile compounds during ripening through the metabolism of triglycerides and proteins [22]. The pasteurization of cows’ milk reduced aromatic intensity and lactic flora, but the addition of donkey milk and freeze-dried *Streptococcus thermophilus* lactic ferments improved the microbiome of Caciotta made from mixed milk and the diversification of flavors in short-ripened cheeses, as also shown in other recent studies [1,7]. In these studies, the addition of *L*. *paracasei* strains was shown to contribute to the development and diversification of flavor in short-ripened cheeses, without affecting the gross composition of the cheese, but with variations in long-chain aldehydes, acids, and esters.

The flavor of milk is highly dependent on low molecular weight volatile compounds that already evaporate at 25 °C and mostly originate from milk fats. These compounds are fat-soluble and bind to the olfactory receptors, stimulating the olfactory sensation [23]. However, the composition of the volatile molecules is influenced by light and heat treatments. In the first study on the flavor of donkey milk [24], a sweet and pleasant taste, a milky aroma, and a sweet flavor were found. These results are due to some chemical properties of donkey milk: a high lactose content, low fat content and its composition [25]. In a study on the volatile compounds profile of donkey milk [23], the presence of 2-heptanone (cheesy and sweet odor), 1,3-bis(1,1-dimethylethyl)-benzen (odor unknown), nonanal (aldehydic and fatty odor), D-limonene (odor of citrus), octanoic acid (fatty and cheesy odor), and 1-octanol (fruity odor) was detected. A sensory analysis by a panel of experts as well as an analysis of the composition of the culturable microbiota can be very useful to improve the quality of Caciotta cheese with added donkey milk. Calasso et al. [26] report that Caciotta cheese is a carrier of probiotic bacteria that survive the manufacturing process and maintain their viability until the end of ripening. Recent studies characterizing the peptides released by the bacterial proteolytic system and their bioactivity in fermented donkey milk also show the presence of peptides with antibacterial, antiviral, and antioxidant properties [13,27,28]. According to recent research, although donkey milk is already a well-established nutraceutical, the LAB fermentation process could increase its value as a fermented functional food [28,29]. Therefore, a Caciotta-type cheese with the addition of donkey milk could also be part of a market for probiotic products.

## 5. Conclusions

Our results show that adding donkey milk to cheesemaking affects some attributes of acceptability. In particular, the acceptability of Caciotta cheese with or without donkey milk was quite different and was also influenced by the ripening time. The mixed Caciotta cheese with a ripening time of 45 days was preferred in terms of color, aroma, flavor and overall liking over the control cheese made with cow’s milk only. Overall, these results are a step towards characterizing cheese made with the addition of donkey milk. The use of donkey milk can lead to a differentiation so as to expand the range of dairy products that are particularly appreciated by consumers interested in innovative products. However, to obtain a more comprehensive characterization, further studies are needed to assess the impact of the addition of donkey milk on the physicochemical composition and sensory profile of the cheese and to identify the choices that most influence consumers’ decisions.

## Figures and Tables

**Table 1:** Chemical parameters of Caciotta cheeses (control cheese, only cow milk) and experimental cheese (5% donkey milk addition) at 10 and 45 days of ripening (mean ± SD).

Parameter	10 Days		*p-*Value	45 Days		*p-*Value
	Cow Cheese	Mixed Cheese		Cow Cheese	Mixed Cheese	
DM, g/kg	566.72 ± 35.0	560.86 ± 34.10	0.845	691.12 ± 41.0	683.97 ± 41.4	0.833
Protein, g/kg	221.38 ± 24.1	218.85 ± 24.30	0.918	246.18 ± 25.6	243.37 ± 25.7	0.755
Fat, g/kg	257.60 ± 28.2	254.26 ± 30.20	0.893	270.30 ± 29.7	266.80 ± 30.8	0.840
Ash, g/kg	36 ± 2.78	32.55 ± 2.8	0.118	31.91 ± 3.1	28.85 ± 2.3	0.241
pH	5.10 ± 0.05	5.00 ± 0.02	0.032	4.93 ± 0.03	4.60 ± 0.01	0.001

**Table 2:** Consumer liking scores (mean ± SD) and comparisons (*p*-Values).

Parameter	Ripening Period				*p-*Value			
	10 Days		45 Days		Cow Cheese vs. Mixed Cheese		10 vs. 45 Ripening Days	
	Cow Cheese	Mixed Cheese	Cow Cheese	Mixed Cheese	10 Days	45 Days	Cow Cheese	Mixed Cheese
Color	7.24 ± 1.30	7.02 ± 1.23	6.76 ± 0.91	7.38 ± 0.96	0.412	0.002	0.041	0.130
Aroma	7.13 ± 1.08	6.64 ± 1.35	6.64 ± 0.96	7.11 ± 0.98	0.051	0.020	0.025	0.064
Flavor	7.27 ± 1.27	6.62 ± 1.40	6.87 ± 1.10	7.51 ± 0.97	0.022	0.004	0.113	0.001
Texture	6.78 ± 1.52	6.89 ± 1.30	7.00 ± 1.33	7.24 ± 0.88	0.711	0.311	0.462	0.132
Overall liking	7.29 ± 1.18	6.71 ± 1.24	6.87 ± 1.01	7.56 ± 0.89	0.031	0.001	0.072	0.001

## Data Availability

The data that support the findings of this study are available from the corresponding author upon reasonable request.
